# Fast and robust optical flow for time-lapse microscopy using super-voxels

**DOI:** 10.1093/bioinformatics/bts706

**Published:** 2012-12-14

**Authors:** Fernando Amat, Eugene W. Myers, Philipp J. Keller

**Affiliations:** ^1^Howard Hughes Medical Institute, Janelia Farm Research Campus, Ashburn, VA 20147, USA and ^2^Max Planck Institute of Molecular Cell Biology and Genetics, 01307 Dresden, Germany

## Abstract

**Motivation:** Optical flow is a key method used for quantitative motion estimation of biological structures in light microscopy. It has also been used as a key module in segmentation and tracking systems and is considered a mature technology in the field of computer vision. However, most of the research focused on 2D natural images, which are small in size and rich in edges and texture information. In contrast, 3D time-lapse recordings of biological specimens comprise up to several terabytes of image data and often exhibit complex object dynamics as well as blurring due to the point-spread-function of the microscope. Thus, new approaches to optical flow are required to improve performance for such data.

**Results:** We solve optical flow in large 3D time-lapse microscopy datasets by defining a Markov random field (MRF) over super-voxels in the foreground and applying motion smoothness constraints between super-voxels instead of voxel-wise. This model is tailored to the specific characteristics of light microscopy datasets: super-voxels help registration in textureless areas, the MRF over super-voxels efficiently propagates motion information between neighboring cells and the background subtraction and super-voxels reduce the dimensionality of the problem by an order of magnitude. We validate our approach on large 3D time-lapse datasets of *Drosophila* and zebrafish development by analyzing cell motion patterns. We show that our approach is, on average, 10 × faster than commonly used optical flow implementations in the Insight Tool-Kit (ITK) and reduces the average flow end point error by 50% in regions with complex dynamic processes, such as cell divisions.

**Availability:** Source code freely available in the Software section at http://janelia.org/lab/keller-lab.

**Contact:** amatf@janelia.hhmi.org or kellerp@janelia.hhmi.org

**Supplementary information:** Supplementary data are available at *Bioinformatics* online.

## 1 INTRODUCTION

**Fig. 1.**
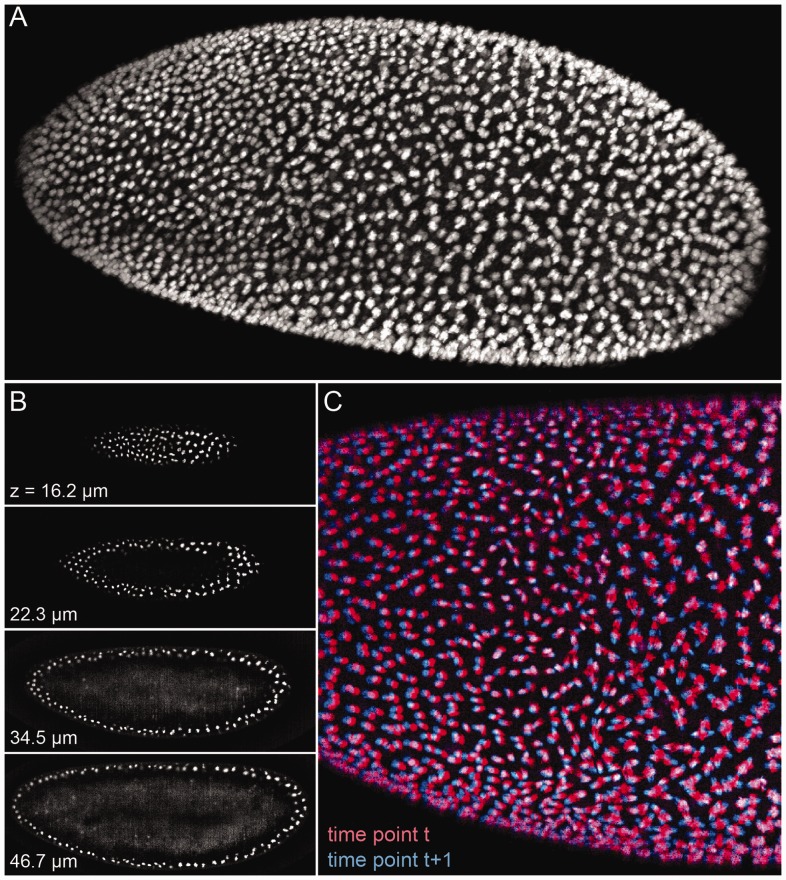
(**A**) Rendering of 3D volume obtained with SiMView light-sheet microscopy (Tomer *et al.*, 2012). Each of the objects represents a single cell nucleus marked by a fluorescent reporter in a *Drosophila* embryo. Dimensions are 602 × 1386 × 110 voxels per volume (0.4 × 0.4 × 2.0 µm^3^ voxel size). The embryo is ∼550 µm long and 200 µm in diameter. (**B**) Optical slices of the volume visualized in (A). (**C**) Enlarged view of two superimposed consecutive time points. Multiple motions, such as cell divisions and cell migration, occur in the same volume

Automated computational techniques are essential for the quantitative analysis of cellular dynamics using time-lapse light microscopy. For example, to quantitatively reconstruct the development of large multi-cellular organisms such as entire *Drosophila* and zebrafish embryos, tens of thousands of cells need to be segmented and tracked at high spatial resolution (McMahon *et al.*, 2008; Tomer *et al.*, 2012) (Fig. 1). Such analyses are of fundamental importance to understanding the development of biological tissues, to reconstructing functional defects in mutants and disease models and to quantitatively dissecting the mechanisms underlying the cellular building plan of entire complex organisms (Keller *et al.*, 2008). However, many computational challenges are encountered when performing key tasks, such as image registration, cell segmentation and cell tracking, in complex microscopy datasets (Khairy *et al.*, 2008; Li *et al.*, 2007; Lou *et al.*, 2011; Preibisch *et al.*, 2010; Rubio-Guivernau *et al.*, 2012).

Optical flow computation is one of the central tasks used to perform quantitative motion estimation of biological structures in time-lapse light microscopy, from the subcellular level to the tissue scale (Abramoff and Viergever, 2002; Buibas *et al.*, 2010; Delpiano *et al.*, 2011; Roberts *et al.*, 2010). Optical flow is defined as the vector field capturing the motion of brightness patterns between adjacent volumes in time (Horn and Schunck, 1981; since our examples are 3D images, we use the term ‘volume’ to refer to the datasets used in optical flow computation. However, our approach and code work also for 2D images). On the cellular level, optical flow information can theoretically be obtained from single-cell tracking data. However, comprehensive and accurate cell tracking in complex multi-cellular organisms is currently an open research problem (Tomer *et al.*, 2012, Lou *et al.*, 2011). Here, optical flow methods can be useful for analyses of group dynamics, which do not require single-cell resolution, or, conversely, as the first module in a larger cell tracking framework. In this latter scenario, the flow information informs the tracking algorithm and helps improving results for regions exhibiting complex or fast cell dynamics.

Optical flow computation has been the object of decades of research, and it is considered a mature technology in many computer vision applications (Baker *et al.*, 2011). However, most approaches have been tested in relatively small 2D natural images, which are dense and rich in edges and texture information. The Middlebury database (Baker *et al.*, 2011) used as a benchmark in the computer vision community is a good example of these types of images. Fluorescence microscopy volumes of biological structures are qualitatively very different from natural images (Fig. 1). They are sparse (in datasets similar to Figure 1, 80–95% of voxels are background; throughout the text, we use the term ‘voxel’ to generically refer to each intensity value in a dataset independent of the dimensionality of the data) and contain relatively textureless objects, which typically appear blurred owing to the point spread function of the microscope and the characteristics of commonly used fluorescent labeling strategies. Moreover, neighboring objects with similar appearance and multiple motions in the same volume are very common. Finally, microscopy volumes tend to be much larger than natural images, which demands computationally efficient approaches. Here, we present a new algorithm for optical flow estimation tailored to large fluorescence light microscopy 3D time-lapse datasets as the one shown in Figure 1. The key idea is to define a model that takes into account the specific characteristics of time-lapse microscopy data. In particular, we define a Markov random field (MRF) over super-voxels to improve registration in textureless areas, propagate motion information efficiently between neighboring structures and speed up computations by reducing the complexity of the problem.

### 1.1 Optical flow techniques

In this paragraph, we highlight some of the fundamental insights introduced over the past few decades. We refer the reader to Baker *et al.* (2011) for a recent comprehensive review. First, Lucas and Kanade (1981) proposed a local approach by solving the optical flow independently in small rectangular regions that partition the entire volume. This approach produces a sparse field because it is ill posed for large regions with uniform appearance. In contrast, Horn and Schunck (1981) introduced a global method, where the flow is calculated at each voxel instead of a rectangular region by introducing smoothness constraints between adjacent voxels as a regularization strategy. This approach produces a dense field, but it cannot resolve motion discontinuities. Black and Anandan (1996) introduced robust metrics, instead of the traditional 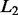 norm, to improve results in motion discontinuity boundaries and regions with intensity changes between volumes. Bruhn *et al.* (2005) merged the benefits of all of these previous approaches in a *combined local-to-global* approach, where a robust Horn and Schunck formulation was solved at different spatial scales, effectively incorporating the benefits of the approach by Lucas and Kanade. Other relevant insights are the application of different weights to each of the smoothness terms to add robustness against motion discontinuities, the detection of occluded regions (Ayvaci *et al.*, 2010) and the application of a smoothing filter to the flow after each iteration of the optimization procedure (Sun *et al.*, 2010; Thirion, 1998) to improve accuracy. Over the years, there has also been progress on real-time optical flow, especially with recent Graphics Processing Unit (GPU) implementations (Werlberger *et al.*, 2009). Unfortunately, software incorporating the most recent advances is not publicly available, and it is not clear whether some of these techniques can be scaled to large 3D datasets according to the timing reported in the benchmarks by Baker *et al.*, (2011).

Most biomedical optical flow applications tend to implement and report results using similar methodologies to the ones explained earlier in the text without tailoring them to the characteristics of the data. For example, Pock *et al.* (2007) presented a total variation (TV)-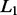 optical flow model for clinical datasets. However, even with the use of image pyramids to solve the problem efficiently, this approach was still slow for large 3D datasets, and it did not always outperform the Insight ToolKit (ITK) implementations. ITK is a multi-threaded C++ library for 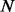-dimensional image registration and segmentation, and it is the most common baseline for comparing the performance and accuracy of new algorithms in the bioimaging domain. Many recent articles use similar strategies to target specifically time-lapse light microscopy datasets (Delpiano *et al.*, 2011; Lombardot *et al.*, 2008; Pizarro *et al.*, 2011), which demonstrate the general interest in applying optical flow to the type of datasets presented in this article. In the following sections, we present an optical flow formulation specifically tailored to solving optical flow for 3D time-lapse microscopy volumes. We show that our method is 10 × faster and reduces the average flow end point error (EE) by 50% for complex dynamic processes, such as cell divisions, with respect to optical flow algorithms available in the ITK library.

## 2 APPROACH

**Fig. 2.**
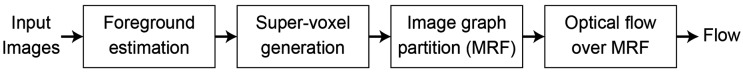
Block diagram representing the pipeline described in this article to estimate optical flow. Optical flow is performed over a set of super-voxels in the volume foreground, and the smoothness constraints are imposed between neighboring (and possibly non-adjacent) super-voxels instead of between connected voxels. This approach guides the registration process of neighboring nuclei with similar dynamics to a better solution than previous approaches

First, we use a conservative foreground/background segmentation to consider only useful pixels. Background removal avoids the optical flow ambiguity in large uniform uninformative regions of the volume and improves computational efficiency. Second, we use a region-based approach to improve performance in the textureless objects. Glocker *et al.* (2008) proposed a similar approach by dividing the image in a rectangular grid. However, as shown in Table 3, rectangular grids do not adapt well to sparse signals and degrade performance, as a single rectangular region can contain two objects with different dynamics. Prinet *et al.* (2006) and Xu *et al.* (2008) also presented region-based approaches to optical flow. However, their segmentation assumptions cannot be applied to light microscopy images owing to the lack of edge and color information. Therefore, we use recent advances in fast super-voxel generation (Achanta *et al.*, 2012) to group flows into small subsets. We combine the foreground/background mask with non-adjacent super-voxel regions to generate a volume partition graph over the set of super-voxels. Then, all smoothness constraints are taken between neighboring super-voxels instead of adjacent voxels, which effectively propagate motion information between close-by cellular structures with similar motions. This graphical model effectively captures specific characteristic of time-lapse light microscopy data. Recently, Gkamas and Nikou (2011) also used super-voxels for optical flow estimation. They added super-voxels to the combined local-to-global framework to establish disconnected motion boundaries between different objects in dense natural images, which is opposite to the strategy in our MRF model for microscopy images, showing that time-lapse microscopy image should be treated differently. Aside from robustness, the model for optical flow presented here allows us to speed up the optimization by an order of magnitude. Finally, we show how standard procedures, such as robust metrics and multi-scale optimization schemes, are also effective in the microscopy imaging domain to improve performance. Our combined framework thus improves and extends optical flow to the application of large-scale time-lapse fluorescence light microscopy images. Figure 2 summarizes the steps described in the next subsections.

## 3 METHODS

Given two 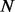-dimensional images of the same size, 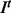 (source volume) and 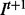 (target volume), our final goal is to estimate a motion field 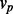 for each voxel 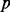 to register the target volume to the source volume.

### 3.1 Image model

When most objects present in the volume are textureless and similar to each other, single voxels are not very informative. In other words, just trying to match single intensities leads to poor solutions. Most optical flow approaches try to guide the registration in textureless areas by imposing a smoothness constraint between adjacent voxels. Unfortunately, microscopy volumes tend to contain many background voxels, which also misguide the smoothness constraint. Thus, we need better partitioning of the volume to improve optical flow.

**Fig. 3.**
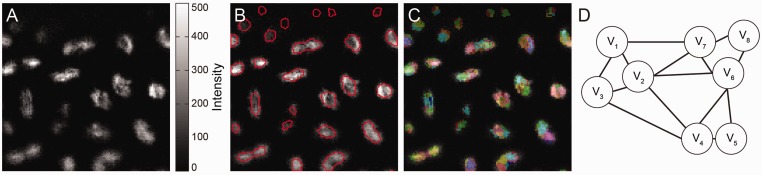
Step for constructing an MRF over the super-voxels on the volume foreground to partition the volume and perform robust optical flow. (**A**) 2D slice of raw data from Figure 1. We show only a slice to simplify the visualization, but the method is implemented in 3D. (**B**) Outline of the foreground mask obtained with a trained classifier in Ilastik (Sommer *et al.*, 2011). Some connected components correspond to multiple nuclei. (**C**) Slice of 3D SLIC (Achanta *et al.*, 2012) super-voxels calculated over the foreground. Super-voxels respect object boundaries of nuclei in the same foreground connected component. (**D**) Edges added between neighboring super-voxels to generate an MRF. Each node *V_i_* represents a super-voxel in panel C. This is the final volume partition model where we perform optical flow. We impose the smoothness conditions over entire super-voxels instead of voxelwise

First, we generate a foreground/background mask (Fig. 3B) to ignore voxels containing no information in the volume. This mask can be as simple as an intensity threshold or any other existing background detection method. Aside from removing non-informative voxels, the mask also helps speed up convergence, as it reduces the number of motion vectors 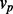 we need to estimate. Data sparsity is problematic and advantageous at the same time, as it precludes the imposition of standard smoothing constraints but it allows a reduction in the size of the problem in the flow calculation.

Once we have a set of foreground voxels, we want to apply the intuition from Xu *et al.* (2008) that region-based optical flow helps in textureless areas. Unfortunately, segmentation techniques tend to be computational costly in large 3D biomedical volumes, and color information is often not available. The connected components of foreground regions contain multiple cells (Fig. 3B), so we cannot use them directly for segmentation. Moreover, cellular structures change shape in a non-rigid manner from one time point to another. Thus, it is not advisable to segment full objects into a single region. Otherwise, the motion model would be too complex. We take advantage of recent advances by Achanta *et al.* (2012) to generate fast super-voxels based on intensity and geometric distance in the volume. Simple linear iterative clustering (SLIC) super-voxels segment each nucleus into a small number of regions while usually respecting the boundaries between different objects (Fig. 3C). Thus, we can expect that all voxels within a super-voxel should have similar motion. Results in Table 3 show that super-voxels outperform fixed-size rectangular regions similar to Lucas and Kanade (1981), as rectangles can sometimes lie in the middle of two objects with different dynamics and degrade performance.

The super-voxels form a partition of the elements in the volume foreground. The final step needed to model the volume is to connect neighboring super-voxels to capture common dynamics between regions. We will define an edge between two super-voxels if their centers of mass are below a distance threshold 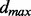. This definition forms an MRF (or equivalently a partition graph) over the foreground voxels (Fig. 3D), where we can directly impose smoothness constraints to calculate optical flow. This setup is necessary because often two regions with coherent dynamics are completely disconnected by background voxels, so traditional voxel-based regularizations are not as effective.

### 3.2 Optimization model

Most approaches in optical flow use the *brightness constancy* assumption
(1)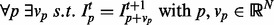 to pose optical flow as the following optimization problem: (2)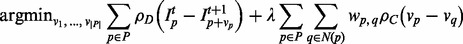 where 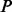 is the set of voxels in the volume, 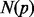 are adjacent neighboring voxels in the volume (using 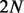 or 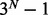 connectivity) and 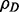 and 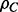 are robust cost functions such as Huber penalty, *L*_1_, TV or Lorentzian (Black and Anandan, 1996). The first sum term in Equation (2) with 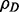 can be considered a unary potential or data term, in which we want to match the intensity between two volumes. In this context, robust metrics are important to allow fluctuations in the volume intensity. However, this term by itself does not offer enough constraints for the motion field 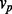. Thus, the second term in Equation (2), referred to as the pairwise potentials or smoothness term, is incorporated to regularize the solution. Here, robust metrics are important to allow for discontinuities in the flow field between different objects in the scene (Black and Anandan, 1996). Finally, it is common to adapt the smoothness term at the pixel level by defining a weight 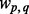 based on edge intensity, effectively reducing the importance of the smoothness constraint in areas of possible motion discontinuities.

Robust metrics alone and voxel-wise smooth flow assumptions are not enough to handle the challenges present in microscopy volumes: given the sparsity, the lack of distinct features between objects and the multiple dynamics in a single volume, the energy terms defined in Equation (2) are not strong enough to guide the optimization process to the right minimum, as shown in Section 4. Using Equation (2) as a model and the MRF over super-voxels constructed in Section 3.1, we can define a new optimization problem: (3)
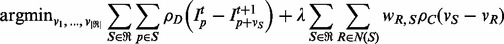

where 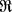 is the set of super-voxels in the graph partition, and we calculate a single translation 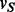 for each region. The modification to the data term helps further regularizing the solution in textureless regions to guide the optimization to the right solution. Moreover, we have reduced the dimensionality of the search by several orders of magnitude (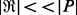). In this case, we decided not to use global affine transformation models, as they do not fit the large variability in cell dynamics. In contrast, we determined experimentally that a local affine model was not necessary to capture those dynamics, so we introduced a compromise with a local translational flow field for each super-voxel. Finally, we adapted the concept from Equation (2) of adaptively adjusting the weight 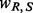 of the smoothness constraint between connected regions in the graph. However, we cannot use edge information because regions may not be adjacent to each other. In our case, we define 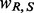 as follows: (4)
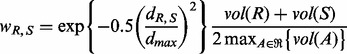

where 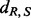 is the distance between the center of masses of super-voxels 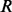 and 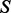, and 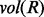 is the number of voxels contained in region 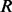. Intuitively, the first term decreases interaction between super-voxels if regions are far apart, and the second term decreases interaction if they do not represent large sets of voxels.

Even with this region-based regularization, the data term is still not powerful enough to always return the right solution (Table 1), as most of the objects in the volume look very similar (Fig. 1). In our case, the term 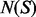 connects entire neighboring regions (not only adjacent voxels), which agrees with the assumption that we have multiple cells with common dynamics in some areas. By connecting non-adjacent super-voxels, the smoothness constraint is imposed much more efficiently over non-connected objects with similar dynamics.

**Table 1.** Stability and importance of parameter *d*_max_ to improve accuracy, for the test region without cell divisions

Method	EE	EE	EE	EE	AUC
	90%ile	95%ile	99%ile	100%ile	
None	0.79	0.89	1.01	2.19	0.77
Our, *d*_max_ = 10	0.13	0.34	1.86	2.27	0.93
*d*_max_ = 25	0.12	0.15	0.39	1.51	0.98
*d*_max_ = 40	0.13	0.16	0.34	0.48	0.97
ITK-demon	0.19	0.29	0.45	0.58	0.97
ITK-curvature	0.41	0.55	0.83	1.34	0.89

Each entry in the table is equivalent to a data point in the plots from Figure 5A. EE *X*% ile indicates the *X*^th^ percentile of the list of EE errors for all nuclei in the ground truth annotation.

Setting the correct value for 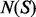 is crucial to achieve good flow estimations. In our case, the size of 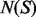 is controlled by the parameter 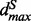, which defines the maximum distance (in voxels) between two region centroids to consider them neighbors or not. Intuitively, we have reduced the complexity of 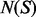 to one parameter per node that controls how global or local we expect object dynamics to be. We can determine an appropriate value for the 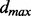 parameter by qualitatively experimenting on different volumes or testing against some ground truth (Sun *et al.*, 2008). Tables 1 and 2 show that it is possible to find a single value that works well across very different motion regimens. However, if the user has a priori information of cell division locations or group motion, it is straightforward to locally set the appropriate 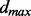 for each region to improve accuracy results.

**Table 2.** Stability and importance of parameter *d*_max_ to improve accuracy, for the test region with cell divisions

Method	EE	EE	EE	EE	AUC
	90%ile	95%ile	99%ile	100%ile	
None	0.93	1.03	1.35	1.47	0.76
Our, *d*_max_ = 10	0.40	0.51	0.76	1.23	0.93
*d*_max_ = 25	0.47	0.56	0.84	1.20	0.92
*d*_max_ = 40	0.48	0.58	0.78	1.08	0.92
ITK-demon	0.86	0.98	1.28	1.39	0.84
ITK-curvature	0.81	0.91	1.28	1.58	0.82

Each entry in the table is equivalent to a data point in the plots from Figure 5B. EE *X*% ile indicates the *X*^th^ percentile of the list of EE errors for all nuclei in the ground truth annotation.

### 3.3 Implementation details

To generate super-voxels, we use the available source code for SLIC super-voxels (Achanta *et al.*, 2012). Achanta *et al.* (2012) is appealing, as we can control the expected size of each super-voxel and its complexity is linear in the number of voxels, making it a reasonable choice for large 3D volumes. Even if the volume consists of grayscale data, the generated super-voxels (Fig. 3C) still respect most object boundaries. Since we have a foreground mask, we tested two approaches: (i) first calculate super-voxels over the entire volume and then apply the mask; or (ii) first apply the mask and then calculate super-voxels only in the foreground. Empirically, both approaches provide similar results, so we use the second approach because it is faster.

To solve the optimization in Equation (3), we use the Limited memory Broyden, Fletcher, Goldfarb and Shanno quasi-Newton method made available by Byrd *et al.* (1994). In particular, 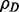 and 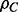 are both defined with the Huber cost function (Huber, 1981). Even though the Huber cost function has a discontinuous second derivative, Li (1995) proved that the function is regular enough to converge using quasi-Newton methods. We use five-point finite difference along each dimension as well as tri-linear interpolation to compute derivatives with subvoxel accuracy at any point in the target volume. We filter the raw data with a small Gaussian (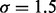) in each direction to smooth the gradient calculations. Finally, as suggested in previous studies, we use a Gaussian pyramid on the volumes to produce a coarse-to-fine solution of the flow. This pyramid not only helps avoiding local minima in the optimization to resolve larger displacements, but also speeds up convergence (Table 3). We also downsample the foreground/background mask and the super-voxels accordingly. All these calculations are performed using a scale parameter along each dimension, as it is common in microscopy volumes to have anisotropic sampling along different axes.

**Table 3.** Resulting accuracy when not using some of the modeling and implementation techniques explained in Sections 3 and 3.3, for the test region with cell divisions (Fig. 4C)

Method	EE	EE	EE	EE	AUC	Time (s)
	90%ile	95%ile	99%ile	100%ile		
None	0.70	0.93	1.03	1.35	0.80	0
Default	0.47	0.56	0.84	1.20	0.92	185
Pyramid levels = 2	0.46	0.57	1.03	1.11	0.92	178
Pyramid level = 1	0.70	1.01	1.37	1.61	0.88	320
*L*_2_	0.51	0.62	0.89	1.21	0.91	181
Voxel-based	0.94	1.05	1.39	1.45	0.81	1754
SLIC step = 3	0.85	0.98	1.31	1.19	0.84	191
SLIC step = 7	0.49	0.61	1.11	1.76	0.92	169
Grid step = 3	0.93	1.04	1.29	1.41	0.83	170
Grid step = 5	0.84	0.97	1.34	1.42	0.86	161
Grid step = 7	0.69	0.78	1.31	1.51	0.88	153
Watershed	0.45	0.55	0.85	1.40	0.92	174

The most significant improvement is obtained by moving from a voxel-based registration to a super-voxel–based registration. However, all elements described in this article improve optical flow accuracy. The default method refers to our method with the parameters defined in Section 4.2. Section 1.3 in the Supplementary Material contains a full description of implementation decisions involved in the deactivation of algorithmic modules for each row in this table.

## 4 RESULTS

We evaluate our approach in scanned light-sheet microscopy datasets. Light-sheet microscopy provides exceptionally high imaging speeds while minimizing the energy load on the biological specimen, and has thus emerged as an essential tool for life sciences. This combination of capabilities is invaluable for live imaging applications and enables quantitative imaging of cellular dynamics throughout the development of complex organisms such as entire *Drosophila* and zebrafish embryos (Fig. 1 and videos in the Supplementary Material). Light-sheet microscopes often produce terabytes of image data per specimen, which need to be analyzed with efficient computational approaches.

We tested our approach in two different biological model systems using previously published datasets of *Drosophila* (Tomer *et al.*, 2012) and zebrafish (Keller *et al.*, 2008). Two videos are included in the Supplementary Material to show the complete results of the optical flow estimation and how it allows analyzing different motion patterns for different groups of cells. Each volume of the *Drosophila* dataset consists of 602 × 1386 × 110 voxels (179 MB in UINT16), and each pair of time points was processed in 3 min with our method (all Central Processing Unit (CPU) running times reported in this article were determined on a workstation with Intel® Xeon® X5690 CPU with 3.47 GHz clock rate). In total, we processed 50 time points (9 GB of data) following a cell division wave in early development.

Each volume of the zebrafish dataset consists of 1064 × 1034 × 500 voxels (379 MB in UINT16), and each pair of time points was processed in 9 min with our method. In total, we processed 220 time points (83 GB of image data) to follow epiboly and the formation of the body axis.

Additional evaluation of the proposed and baseline methods using synthetic data is provided in the Supplementary Material. We simulate fluorescent nuclei images with different types of motion (linear, cell division and Brownian), different signal-to-noise ratios, different cell densities and different photobleaching settings to show that our method is applicable to different types of fluorescence microscopy techniques and cell dynamics.

### 4.1 Baseline and ground truth

We compare our results with two common implementations of optical flow for 3D biomedical volumes available in the ITK (Ibanez *et al.*, 2003). Lombardot *et al.* (2008) discussed these implementations in the context of time-lapse light microscopy for organism development at single-cell resolution. In particular, we use the multi-scale ITK-demon optical flow, which implements a multi-scale version of Thirion’s demon algorithm (Thirion, 1998), as our first baseline. The algorithm has complexity 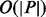, where 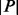 is the number of voxels in the volume, and solves Equation (2) with 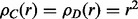. The second baseline is a modification of the ITK-demon algorithm using regularization of the second derivative of the flow instead of the first order, which has been shown to provide better convergence properties for certain types of volumes (Fischer and Modersitzki, 2004). This algorithm has complexity 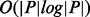, and we will refer to it as ITK-curvature throughout the text. Both implementations are written in C++ using multi-threaded and multi-scale techniques for efficient handling of large biomedical datasets.

**Fig. 4.**
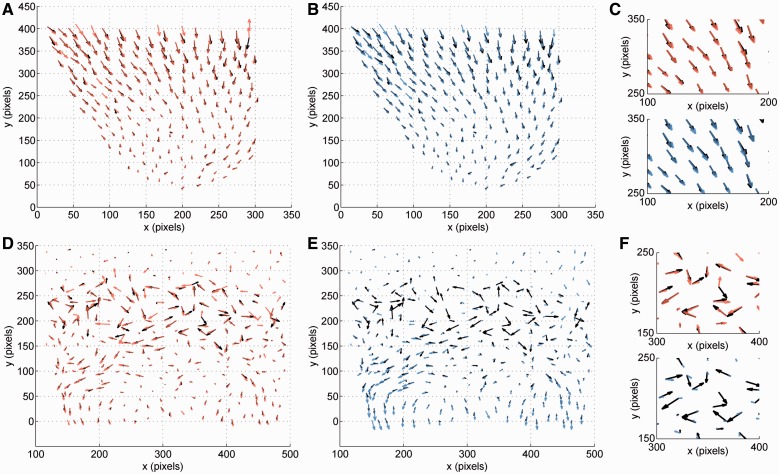
(**A**) Motion field (black: ground truth, red: estimate by our approach) projected on the *X–Y* plane for a subregion of the volume in Figure 1 with smooth flow. Each arrow corresponds to a nucleus centroid. (**B**) Same as (A) for motion field estimated by the baseline method multi-scale ITK-demon (blue). (**C**) Enlarged subregion of (A) and (B). (**D**) Same as (A) for a subregion where cells are dividing, which translates into non-smooth dynamics for neighboring nuclei. Our approach is still able to predict the correct motion for 99% of the nuclei. Supplementary Movie S1 shows the raw data and the output of our optical flow algorithm side by side for the entire time series. (**E**) Same as (B) for the subregion presented in (D). The complex dynamics complicate setting a global motion smoothing parameter that works for all nuclei at the same time. (**F**) Enlarged subregion of (D) and (E). Most of the ITK flow (blue) results as zero because it cannot adapt to the complex motion pattern

To quantitatively assess performance, we manually segmented nuclei in two different regions of adjacent time points in the *Drosophila* dataset using the software package Imaris (Bitflow). Each region represents different dynamic regimens (Fig. 4). We then manually assigned correspondences between segmented nuclei to calculate the displacement (Fig. 4). Given that the nuclei are textureless, we cannot assign unique voxel-to-voxel correspondences, and thus, our ground truth evaluates center of mass displacement for each nucleus. We use the flow EE metric (5)
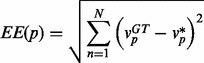
 defined by Otte and Nagel (1994) to measure accuracy. Here 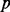 is the center of mass for a given nucleus, 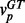 is the ground truth flow at centroid 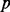 and 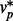 is the estimated flow for each individual algorithm. 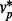 is estimated as the mean flow of all voxels contained within the segmentation mask for each nucleus in the ground truth. Because a nucleus is usually split in several super-voxels, this estimation can be seen as a weighted average of the calculated optical flow for each super-voxel proportional to its size. Section 1.1 in the Supplementary Material contains statistics on the accuracy of the ground truth 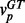.

**Fig. 5.**
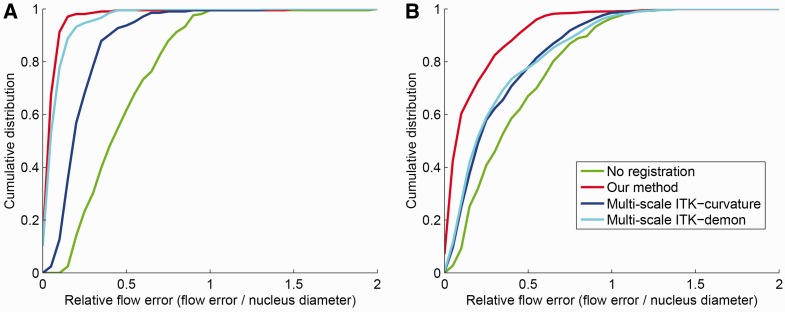
Optical flow results for light-sheet microscopy using different methods. See text for details on ground truth definition. *X*-axis represents the EE for each nucleus centroid normalized by the equivalent diameter of each nucleus. As a rule of thumb, values < 0.5 are considered good for most quantitative applications, whereas values > 1.0 are not good. Values between 0.5 and 1.0 are acceptable, but flow tracking has a higher error rate. Method labeled as ‘None’ represents the original displacement without flow estimation. Panel A shows results on data from Figure 4A and B. Panel B shows results on data from Figure 4D and E. Our method improves accuracy over all baselines in both scenarios, on average, by 23%

Once we have 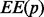 for all nuclei, we can compute different statistics to compare accuracy of different methods. Figure 5 displays the full cumulative distribution of errors, while Tables 1, 2 and 3 display different figures of merit, summarizing the information in the cumulative distribution. In particular, we show several percentiles of the 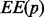 distribution and the area under the curve (AUC). This last figure of merit is typically used in computer vision applications with precision-recall curves, as it summarizes the entire distribution in a single number. We normalize the maximum AUC to 1 to simplify the comparison.

### 4.2 Results in light-sheet microscopy data

For the purpose of a quantitative performance analysis, we selected two regions from two consecutive time points in the *Drosophila* dataset and performed a ground truth annotation for both of them. Figure 5A shows comparative results for the first test region between time points 39 and 40. This region comprises 214 cells with an average diameter of 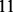 voxels moving all in the same direction, although at different speeds. In this example, the motion between cells is coherent, and thus, smoothness constraints are sufficient in most voxels to compensate for lack of texture. In this case of simple dynamics, our method has an average 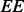 of 0.07, whereas the best ITK method has an average 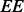 (normalized by nuclei diameter) of 0.10. However, tested on the same hardware, our implementation is consistently 10 × faster. In particular, it takes 3 min to converge for each 3D volume, whereas both ITK algorithms require ∼30 min for the same task. One of the main reasons for the speed improvement is the dimensionality reduction achieved with super-voxels. As an example, in this particular stack, there were 1 117 920 foreground voxels, which resulted in 19 274 super-voxels, reducing the size of the optimization problem ∼60-fold.

Figure 5B shows a very different scenario from the same stack: in this part of the embryo, nuclei are synchronously dividing, and the motion field transitions very rapidly from smooth to non-smooth. In total, we performed a ground truth annotation for 309 cells with an average diameter of 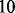 voxels between time points 38 and 39. In this case, our method has an average 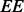 (normalized by nuclei diameter) of 0.16, and the best ITK method has an average 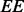 of 0.32. Figure 4A and Table 2 also show that ∼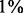 of the nuclei are assigned to the wrong location using our method (Supplementary Fig. S1 shows an enlarged view of the location exhibiting the largest error). This error is due to the fact that neighboring nuclei divide synchronously and two daughters from different mother cells collide, causing the MRF to pull one of them to the wrong location. This region of the volume pushes the limits of optical flow, as touching neighboring objects do not have a coherent motion and suffer displacements larger than the object size.

Tables 1 and 2 show the stability of parameter 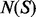 in Equation (3). The accuracy results change gradually with the value of 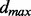, and this allows us to use the same value for all regions and still outperform other approaches. The only exception is 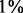 of the nuclei in the first test region, which need an increase in the smoothness constraint to be guided to the correct location, especially at the edges of the MRF (Fig. 4A). In our case, we used 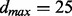 voxels for both the *Drosophila* and zebrafish dataset, which is slightly more than the expected nearest neighbor distance between adjacent nuclei (23 isotropic voxels). This result indicates that, in general, superior results are obtained by directly considering motion information between neighboring cells in the smoothness term, which cannot be achieved with the usual pixel-wise regularization approaches. However, Table 2 also shows that in extreme cases of incoherent motion, such as during cell division, we could benefit from reducing 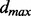 to 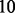 voxels. In this particular case, a cell division detector (Huh *et al.*, 2011) could be used to detect such events and locally adjust the value of 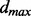. Supplementary Tables S1 and S2 in the Supplementary Material present a more detailed analysis by decomposing the accuracy results in Table 2 between dividing and non-dividing nuclei. An extended accuracy analysis using synthetic data is provided in the Supplementary Material, which further supports the conclusions of this section.

Table 3 shows that all elements introduced in Sections 3 and Section 3.3 are necessary to obtain the best accuracy and performance. In particular, a region-based (SLIC super-voxels in our case) and a multi-scale approach (of at least two levels) are critical to define an appropriate data term and to avoid local minima in Equation (3), respectively. Moreover, the use of super-voxels that adapt to the sparse signal instead of fixed-size rectangular-like regions [as suggested by Glocker *et al.* (2008)] improves accuracy as long as the super-voxels have a minimum size. As the table entry using watershed shows, any oversegmentation method producing reasonable super-voxels adapted to the sparse data could be used within this framework.

All results discussed in this section were obtained with fixed parameters. For our method, we use 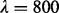, three levels in the pyramid and 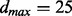. For Huber penalty, we use 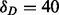, which indicates intensity values are well preserved between frames, and 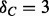. Finally, for the SLIC super-voxels, we use 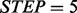 and 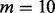 [see Achanta *et al.* (2012) for details]. For both ITK implementations, we performed an optimal parameter search using the ground truth to obtain the best performance. Additionally, we use three pyramid levels for their multi-scale scheme and applied the foreground mask filter for a fair comparison. Finally, we tested ITK algorithms on the raw stacks and on cubic interpolated stacks to generate isotropic sampled voxels to confirm that anisotropy in the data along the *z*-axis was not compromising performance. The final results (data not shown) were undistinguishable, so we performed all comparisons with the anisotropic data because execution time was shorter.

## 5 DISCUSSION

We developed and tested a new model for optical flow tailored to microscopy volumes, in which a large fraction of the objects are textureless and similar in appearance. Moreover, the information in the volume tends to be sparse because many voxels do not contain any information and cellular dynamics can be very variable. A key idea in our approach is to generate a volume partition graph over the foreground voxels, and to perform optical flow directly on that model instead of computing it at the voxel level. This model is tailored to the specific characteristics of time-lapse light microscopy datasets, as it provides the regularization needed to solve optical flow robustly for these types of volumes. At the same time, our method reduces the complexity of the problem by an order of magnitude, which is an invaluable advantage when working with large 3D datasets.

In Section 4.1, we showed that the method might fail in some extreme cases for ∼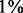 of the nuclei, when neighboring nuclei move in opposite directions. In those scenarios, we are left only with the data term to determine the correct flow. Thus, a possible future direction would be to use different features or point descriptors in the volume intensity to increase robustness of the data term (Brox and Malik, 2011; Liu *et al.*, 2008). It is also possible to constrain the flow field to a diffeomorphism, as two objects cannot originate from the same source point. Finally, if a faster implementation is required, it is straightforward to parallelize the computation of the data term in Equation (3) for each super-voxel using GPU technology. At the moment, this operation takes ∼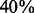 of the time for each function evaluation in the quasi-Newton method, and it is thus a primary candidate for code optimization.

## Supplementary Material

Supplementary Data

## References

[bts706-B1] Abramoff MD, Viergever MA (2002). Computation and visualization of three-dimensional soft tissue motion in
the orbit. IEEE Trans. Med. Imaging.

[bts706-B2] Achanta R (2012). SLIC superpixels compared to state-of-the-art superpixel
methods. IEEE Trans. Pattern Anal. Mach. Intell..

[bts706-B3] Ayvaci A (2010). Occlusion detection and motion estimation with convex
optimization. NIPS'10.

[bts706-B4] Baker S (2011). A database and evaluation methodology for optical flow. Int. J. Comput. Vis..

[bts706-B5] Black MJ, Anandan P (1996). The robust estimation of multiple motions: parametric and piecewise-smooth
flow fields. Comput. Vis. Image Underst..

[bts706-B6] Brox T, Malik J (2011). Large displacement optical flow: descriptor matching in variational motion
estimation. IEEE Trans. PAMI.

[bts706-B7] Bruhn A (2005). Lucas/Kanade meets Horn/Schunck: combining local and global optic flow
methods. Int. J. Comupt. Vis..

[bts706-B8] Buibas M (2010). Mapping the spatiotemporal dynamics of calcium signaling in cellular neural
networks using optical flow. Ann. Biomed. Eng..

[bts706-B9] Byrd RH (1994). A limited memory algorithm for bound constrained
optimization. SIAM J. Sci. Comput..

[bts706-B10] Delpiano J (2011). Performance of optical flow techniques for motion analysis of fluorescent
point signals in confocal microscopy. Mach. Vis. Appl..

[bts706-B11] Fischer B, Modersitzki J (2004). A unified approach to fast image registration and a new curvature based
registration technique. Linear Algebra Appl..

[bts706-B12] Gkamas T, Nikou C (2011). Guiding optical flow estimation using superpixels. International Conference on Digital Signal Processing.

[bts706-B13] Glocker B (2008). Dense image registration through MRFs and efficient linear
programming. Med. Image Anal..

[bts706-B14] Horn BKP, Schunck BG (1981). Determining optical flow. Aritifical Intell..

[bts706-B15] Huber PJ (1981). Robust Statistics.

[bts706-B16] Huh S (2011). Automated mitosis detection of stem cell populations in Phase-Contrast
microscopy images. IEEE Trans. Med. Imaging.

[bts706-B17] Ibanez L (2003). The ITK Software Guide: The Insight Segmentation and Registration Toolkit.

[bts706-B18] Keller PJ (2008). Reconstruction of zebrafish early embryonic development by scanned light
sheet microscopy. Science.

[bts706-B19] Khairy K (2008). Detection of deformable objects in 3D images using Markov-Chain monte carlo
and spherical harmonics. MICCAI.

[bts706-B20] Li G (2007). 3D cell nuclei segmentation based on gradient flow tracking. BMC Cell Biol..

[bts706-B21] Li W (1995). Numerical estimates for the huber M-Estimator problem. Approximation Theory.

[bts706-B22] Liu C (2008). SIFT flow: dense correspondence across different scenes. ECCV.

[bts706-B23] Lombardot B (2008). Evaluation of four 3d non rigid registration methods applied to early
zebrafish development sequences. MIAAB MICCAI.

[bts706-B24] Lou X (2011). Deltr: digital embryo lineage tree reconstructor. 2011 IEEE International Symposium on Biomedical Imaging: From Nano to
Macro.

[bts706-B25] Lucas BD, Kanade T (1981). An iterative image registration technique with an application to stereo
vision. IJCAI.

[bts706-B26] McMahon A (2008). Dynamic analyses of drosophila gastrulation provide insights into
collective cell migration. Science.

[bts706-B27] Otte M, Nagel H (1994). Optical flow estimation: advances and comparisons. ECCV.

[bts706-B28] Pizarro L (2011). Towards dense motion estimation in light and electron
microscopy. ISBI.

[bts706-B29] Pock T (2007). A duality based algorithm for TV-L1-optical-flow image
registration. MICCAI.

[bts706-B30] Preibisch S (2010). Software for bead-based registration of selective plane illumination
microscopy data. Nat. Methods.

[bts706-B31] Prinet V (2006). MRF modeling for optical flow computation from multi-structure
objects. ICIP.

[bts706-B32] Roberts T (2010). Estimating the motion of plant root cells from in vivo confocal laser
scanning microscopy images. Mach. Vis. Appl..

[bts706-B33] Rubio-Guivernau JL (2012). Wavelet-based image fusion in multi-view three-dimensional
microscopy. Bioinformatics.

[bts706-B34] Sommer C (2011). Ilastik: interactive learning and segmentation toolkit. ISBI.

[bts706-B35] Sun D (2008). Learning optical flow. ECCV.

[bts706-B36] Sun D (2010). Secrets of optical flow estimation and their principles. CVPR.

[bts706-B37] Thirion J (1998). Image matching as a diffusion process: an analogy with maxwell’s
demons. Med. Image Anal..

[bts706-B38] Tomer R (2012). Quantitative high-speed imaging of entire developing embryos with
simultaneous multiview light-sheet microscopy. Nat. Methods.

[bts706-B39] Werlberger M (2009). Anisotropic Huber-L1 optical flow. BMVC.

[bts706-B40] Xu L (2008). A segmentation based variational model for accurate optical flow
estimation. ECCV.

