# Supplementary material for: Fast and robust optical flow for time-lapse microscopy using super-voxels
Source: Bioinformatics. 2012 Dec 14;29(3):373–80. doi: 10.1093/bioinformatics/bts706 (PMC3562071; doi:10.1093/bioinformatics/bts706)
Supplement: Supplementary Data [file supp_29_3_373__index.html]

Fast and robust optical flow for time-lapse microscopy using super-voxels — Fast and robust optical flow for time-lapse microscopy using super-voxels — Supplementary Data 

# Fast and robust optical flow for time-lapse microscopy using super-voxels

## Supplementary Data

files

**Files in this Data Supplement:**

- Supplementary Data - pdf file
- Supplementary Data - mov file
- Supplementary Data - mov file
- Supplementary Data - zip file
