# Supplementary material for: Fast and robust optical flow for time-lapse microscopy using super-voxels
Source: Bioinformatics. 2012 Dec 14;29(3):373–80. doi: 10.1093/bioinformatics/bts706 (PMC3562071; doi:10.1093/bioinformatics/bts706)
Supplement: Supplementary Data [file supp_bts706_Supplement.pdf]

BIOINFORMATICS

Vol. 00 no. 00 2012
Pages 1–3

Fast and robust optical flow for time-lapse microscopy using super-voxels (Supplementary Material)

Fernando Amat [1,*], Eugene W. Myers [2] and Philipp J. Keller [1,*]

[1]Howard Hughes Medical Institute, Janelia Farm Research Campus, Ashburn, Virginia, USA
[2]Max Planck Institute of Molecular Cell Biology and Genetics, Dresden, Germany

Received on XXXXX; revised on XXXXX; accepted on XXXXX

Associate Editor: XXXXXXX

Here, we provide additional information related to the results discussed in the main text.

1 RESULTS IN LIGHT SHEET MICROSCOPY DATA

In this section, we present an extension of the results discussed in section 4.2 of the main text. In particular, we extend Tables 1 and 2 in order to present further analyses of our method in different scenarios.

1.1 Accuracy of ground truth in real data

In order to estimate the intrinsic variability in the optical flow ground truth of real data, a second user repeated the process explained in Section 4.1 for a subset of 200 nuclei that had already been segmented for use as ground truth in different subregions. Based on this common annotation of the flow we can estimate statistics for v_p^{GT}. In particular, approximating the uncertainty by a normal distribution, the mean is $\mu = (-0.006, -0.017, -0.002)$ pixels and the standard deviation is $\sigma = (0.161, 0.173, 0.050)$ pixels for each of the 3D directions (uncertainty is uncorrelated between different directions). The uncertainty is so low because the software package Imaris (bitflow) uses a local region growing algorithm to segment each nucleus after the user has clicked on its center. Thus, the center of mass for each nucleus used to calculated optical flow is a stable measurement.

Using these statistics we can run a Monte Carlo simulation to establish confidence intervals of our results. The 99% confidence interval for the AUC obtained from a Monte Carlo simulation using 10000 samples for the graphs presented in Fig. 5 is ± 0.0005. Thus, uncertainty in our ground truth is negligible and our improvement in optical flow accuracy versus the baseline ITK methods is statistically significant.

1.2 Cell division in Fig. 4D

Tables S1 and S2 show a decomposition of the accuracy results presented in the main text for Table 2 based on dividing (24 out of 309) versus non-dividing cells (285 out of 309). The tables clearly show that optical flow is more difficult to estimate when cells are dividing. Moreover, Fig. 4 in the main text also shows that the complexity of cellular dynamics does not relate to cell divisions: there is a spatial transition region from linear dynamics to dividing cells to cells that have just divided and errors accumulate in all regions (especially for the ITK baseline methods) since it is hard to set a global motion smoothing parameter that works in all scenarios at the same time.

Method	EE 90%ile	EE 95%ile	EE 99%ile	EE 100%ile	AUC
Our $d_{max} = 10$	0.52	0.91	1.23	1.23	0.85
$d_{max} = 25$	0.68	0.88	1.20	1.20	0.82
$d_{max} = 40$	0.70	0.87	0.98	0.98	0.83
ITK-demons	1.00	1.15	1.30	1.30	0.64
ITK-curvature	0.91	0.94	1.01	1.01	0.68

Table S1. Accuracy results presented in Table 2 in the main text considering only dividing cells (24 out of 309) in subregion shown in Fig. 4C.

Method	EE 90%ile	EE 95%ile	EE 99%ile	EE 100%ile	AUC
Our $d_{max} = 10$	0.34	0.50	0.66	0.77	0.95
$d_{max} = 25$	0.43	0.55	0.76	1.08	0.93
$d_{max} = 40$	0.45	0.57	0.75	1.08	0.93
ITK-demons	0.81	0.95	1.27	1.39	0.86
ITK-curvature	0.78	0.90	1.29	1.58	0.83

Table S2. Accuracy results presented in Table 2 in the main text considering only non-dividing cells (285 out of 309) in subregion shown in Fig. 4C. Optical flow accuracy is better for non-dividing cells.

1.3 Relevance of different algorithmic elements

In this section we explain the terminology presented in Table 3 and detail which algorithmic elements were suppressed for each entry in the column entitled ”Method”.

- *None*: refers to results in the absence of optical flow computation. Any optical flow methods should perform better than this baseline.

*to whom correspondence should be addressed

© Oxford University Press 2012.

- *Default*: refers to the results using our method with all elements described in Section 2. In particular, this entry is identical to entry "Our, d_{max} = 25" in Table 2.
- *Pyramid levels* p: refers to the results using our method with a different number of levels in the Gaussian pyramid to produce a coarse-to-fine solution. In particular, p equal to 1 corresponds to the deactivation of coarse-to-fine approach.
- L_2: refers to the results of our method when we do not use robust statistics for the cost functions ρ_D and ρ_C. In other words, we use the usual least-squares (or L_2 norm) cost function instead of the Huber penalty.
- *Voxel based*: refers to the results of our method when we do not use a region-based approach to construct the MRF. Instead, we construct the MRF between individual adjacent voxels.
- *Grid step* m: refers to the result of our method when we defined super-voxels as simple square regions of side length m. Thus, instead of adjusting regions to the data we just divide the image *a priori* into a square grid to create regions.
- *SLIC step* s: refers to the result of our method when we change the parameter STEP in the SLIC super-voxels code. This parameter sets an expected value for the size of each super-voxel.
- *Watershed super-voxels*: refers to the results of our method when we generate super-voxels using a watershed approach instead of the SLIC methodology.

All tests were run with the default values for each parameter defined at the end of section 4.2 (if that parameter was applicable).

1.4 Supplementary movies

We provide two movies to show the raw data and the results of our optical flow algorithm side by side. The movies comprise several hundred time points of microscopy data for two different species.

Movie S1 shows the optical flow calculations for *Drosophila* embryonic development during mitotic cycle 13. The volume at each time point consists of 602x1386x110 voxels and 50 time points were processed in total (9 GB of image data). Fig. 1 in the main text shows more details on this dataset. Left panel shows a maximum intensity projection (MIP) of the raw microscopy data. Right panel shows estimated optical flow for each voxel contributing to the MIP. The data is 3D+time and all calculations were performed on the raw data. MIPs are shown to simplify visualization of the results. For each MIP-relevant voxel in the dataset the 3D vector flow (u,v,w) is projected onto the tangent plane of a cylinder to obtain ($d\theta$,dz) (the radial component is negligible). The color map shown in the upper part of the video is used to visualize these components. Two fundamentally different types of movements are present during this time sequence: first, nuclei divisions, in which the parent cell is segmented into two colors by the optical flow, since the two daughter cells move in opposite directions; second, a fast global wave across the embryo initiated by the nuclear divisions, in which all nuclei move collectively.

Movie S2 shows the optical flow calculations for zebrafish development during epiboly and the formation of the body axis. The volume at each time point consists of 1064x1034x500 voxels and 220 time points were processed in total (83 GB of image data). Left panel shows a maximum intensity projection (MIP) of the raw data. Right panel shows estimated optical flow for each voxel contributing to the MIP. The data is 3D+time and all the calculations were performed on the raw data. MIPs are shown to simplify visualization of the results. For each MIP-relevant voxel in the dataset the 3D vector flow (u,v,w) is projected onto the tangent space of a sphere to obtain ($d\theta$,$d\psi$) (the radial component is not predominant in this scenario). The time series includes global cell movements during epiboly (from the animal pole to the vegetal pole, i.e. from top to bottom) as well as convergence movements (towards the midline) underlying the formation of the body axis of the embryo. The optical flow captures both the specimen-intrinsic dynamic behavior (cell migration, cell divisions) and the overall drift of the specimen in the microscope (high-frequency changes in optical flow intensity).

2 RESULTS IN SYNTHETIC DATA

In this section we repeat the previous accuracy analyses of real light sheet microscopy data using synthetic data. The goal is to study the performance of our approach and the two ITK baseline methods in different scenarios related to different fluorescent microscopy techniques and different cell dynamics. In particular, we simulate four main parameters: signal-to-noise ratio (SNR), cell density, cell motion (linear, cell division and Brownian) and photobleaching. Fig. S1 shows synthetic data compared side to side with real light sheet microscopy data (as shown in Fig. 1).

2.1 Simulation

Each nucleus is considered a 3D ellipsoid with 9 parameters: center (x, y, z), main semi-axis length (a, b, c), intensity, type of motion and presence of photobleaching. For all simulations (a, b, c) are independent and identically distributed (i.i.d.) normal random variables with mean 3 pixels and standard deviation 1 pixel. Thus, the average nucleus diameter is 6 pixels. For each experiment we assign a targeted SNR value, which determines the expected intensity value of each pixel based on the $\sqrt{N}$ rule characteristic of shot noise. We perturb at random the expected intensity value of each nuclei with a standard deviation of a tenth of the expected value in order to simulate variability in the fluorescent labeling. If photobleaching is activated in the simulation, the intensity is reduced from one time point to the next by a uniform random value between 40% and 60%.

Each cell is also assigned one of three possible kinds of motion: linear, cell division or Brownian. In the linear case, there is a single global displacement vector that would be applied to a group of neighboring cells in order to simulate coherent group dynamics. The direction of the displacement is chosen at random and the magnitude of the displacement is drawn from a normal distribution with mean 4 pixels ($2/3$ of the expected nuclei diameter) and standard deviation of 0.5 pixels. If a nucleus is labeled dividing we perform the following steps: first, we choose the axis of division at random (this axis is different for each dividing cell). Second, we create two nuclei with a volume equal to half the mother nucleus volume (reflecting different chromatin compaction states). The magnitude of the displacement is drawn from a normal distribution with a mean value of 2 pixels and standard deviation of 0.25 pixels. The intensity of each daughter nucleus is the same as that of the mother nucleus. In the Brownian case, the nucleus is just displaced by drawing three times (one for each coordinate in 3D) from a normal distribution with zero mean and standard deviation of 2 pixels (a $1/3$ of the expected diameter).

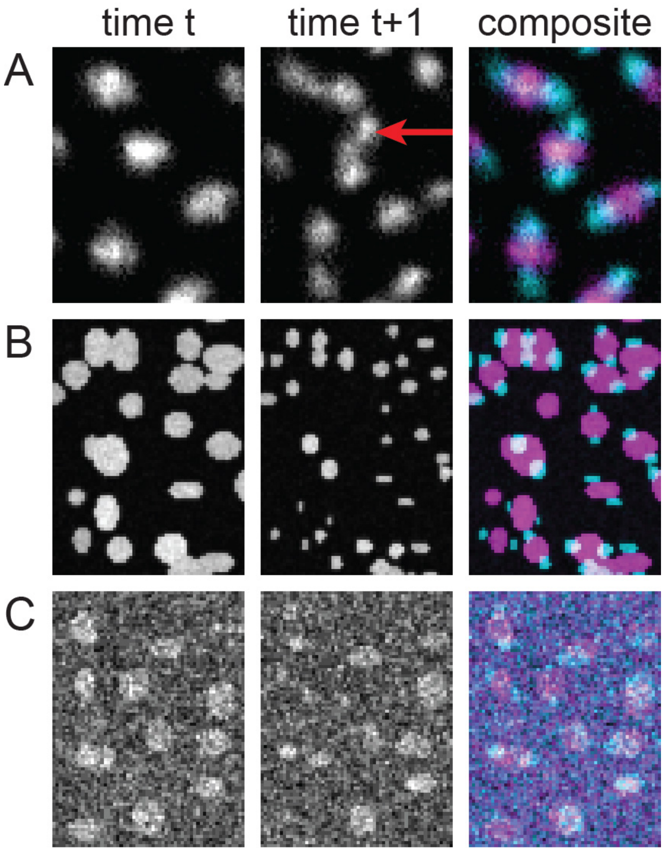

Fig. S1. (A) Enlarged section of a 2D slice of the real microscopy data presented in Fig. 1 in the main text and analyzed for optical flow in Fig. 4 using ground truth. Red arrow points to the nucleus of daughter cells from a recent division event, where the optical flow introduces a major mistake since it is linked in the MRF with the other daughter cell above it from a different mother cell. (B) 2D slice of simulated data for SNR$=10$, cell density $=1.5$, no photobleaching and all dividing cells. Some daughter cells are occluded by other cells, since the underlying division occurred along the z-axis. (C) 2D slice of simulated data for SNR$=2$, cell density $=2$, no photobleaching and cells with three types of motion (linear, Brownian and cell division). All panels represent an 80x60 pixels image patch. In the "composite" column magenta represents the image in time t and cyan represents the image at time $t+1$.

In order to place each nucleus we generate a grid across the image in which objects are equally spaced according to the cell density parameter. For example, if the cell density is 1.5, the expected distance between two adjacent nuclei centroids is 1.5 times the expected diameter (6 pixels). After the grid of nuclei is laid out, we perturb each position with a normal distribution with a mean value of zero and a standard deviation of 4 pixels in each direction.

Finally, we run a collision checker to make sure any two nuclei are not overlapping in space. If that is the case, we keep perturbing the position until there are no occlusions. Once all nuclei have been placed in both time points, we add the following elements to simulate image acquisition properties. First, we add a background intensity of 10 to each pixel in the image. Second, we downsample the image in the Z direction by a factor of 1:5 and blur the image with a Gaussian kernel of sigma 1 pixel to simulate an anisotropic point-spread-function of the microscope. Finally, we add Poisson noise to every voxel in the image with lamba equal to the intensity value in each voxel in order to simulate shot noise.

2.2 Validation

Fig. S1 shows real light sheet microscopy data and our simulated data in order to demonstrate that synthetic data can be used to verify the algorithm. In particular, the light sheet microscopy data has an SNR greater than 10, a cell density of 1.5 and does not present severe photobleaching. As explained in the main text, it presents mainly linear and cell division motions depending on the subregion. The main noticeable difference between the real data and the synthetic data is that the expected nuclei diameter is half in the latter. This characteristic is useful to predict perfomance in later time points, when nuclei sizes tend to decrease. Fig. S2A and S2B are almost equivalent to Fig. 5A and B, which also validates the use of synthetic data. The main difference is in the performance of the ITK-curvature baseline, which in the case of synthetic data is severely degraded due to the sensitivity of the parameters to a change of data properties.

2.3 Accuracy results

All accuracy results shown in Fig. S2 were obtained using the same parameters described in the main text for each algorithm. In other words, we did not optimize the parameters for each scenario, but rather evaluate synthetic data using the optimal parameters obtained from our real data.

The simulations assess that our method outperforms both ITK baselines in almost every possible scenario. The only exception occurs for very low SNR (Fig. S2C), where supervoxels do not perform a meaningful oversegmentation of the nuclei and a gradient descent optimization using derivatives on the images might not converge in the right direction. Moreover, the expected background intensity is 10 counts and the expected nucleus intensity is 14 counts. Once we add shot noise and PSF blurring, matching nuclei to background or to another nuclei has a similar cost in Eq. (2).

Another difference between simulations and real data is a change of the optimal d_{max}. Since the expected nucleus diameter is almost half (11 pixels for real data and 6 pixels for synthetic data) and the cell density is estimated relative to the nuclei diameter, this adjustment of the parameter d_{max} is expected in order to adjust the MRF to the data. This result confirms the conclusions in the main text. As a rule of thumb, d_{max} should be set to the expected nearest neighbor distance between nuclei. However, note that the accuracy degradation for different values of d_{max} is slow enough to allow multiple value of d_{max} to be close to optimal.

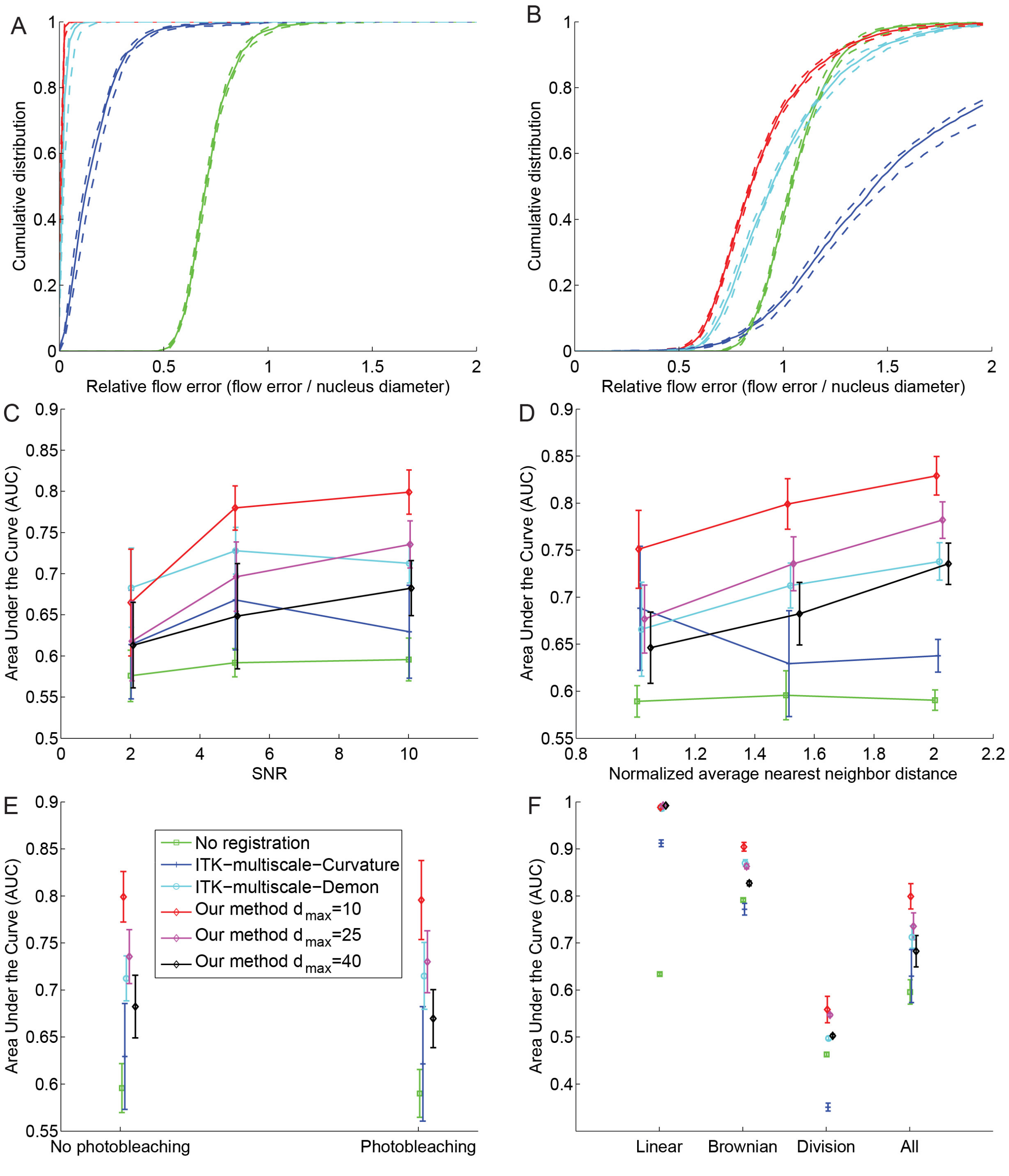

Fig. S2. Summary of accuracy results for optical flow in synthetic data in different conditions. Each test was run 8 times to obtain error bars (dashed lines in panels A and B). (A) Same curves as in Fig. 5 in the main text for synthetic data using SNR= 10, cell density = 1.5, no photobleaching and only linear movements. The data conditions and accuracy results are very similar to the ones shown in Fig. 4A. (B) Same curves as in Fig. 5 in the main text for synthetic data using SNR= 10, cell density = 1.5, no photobleaching and only cell divisions. The data conditions and accuracy results are very similar to the ones shown in Fig. 4B although the shape changes during cell divisions are more extreme in our simulations (Fig. S1), which displaces the curves to the right and produces worse results for the baseline method ITK-multiscale-Curvature. (C) Accuracy results for different SNR using cell density = 1.5, no photobleaching and all 3 motions (linear, Brownian and cell division). (D) Accuracy results for different cell densities using SNR = 10, no photobleaching and all 3 motions. (E) Accuracy results with photobleaching effects using SNR = 10, cell density = 1.5 and all 3 motions. On average, accuracy results are comparable. However, photobleaching widens the error bars. (F) Accuracy results for different motions using SNR = 10 cell density = 1.5 and no photobleaching. For simple linear motions most methods perform almost perfectly while our method clearly outperforms the baseline ITK methods in more complex dynamics. "All" refers to a dataset containing $1/3$ of nuclei with linear dynamics, $1/3$ with Brownian and $1/3$ with divisions.
