# Supplementary figures and images for: Fast and robust optical flow for time-lapse microscopy using super-voxels
Source: Bioinformatics. 2012 Dec 14;29(3):373–80. doi: 10.1093/bioinformatics/bts706 (PMC3562071; doi:10.1093/bioinformatics/bts706)

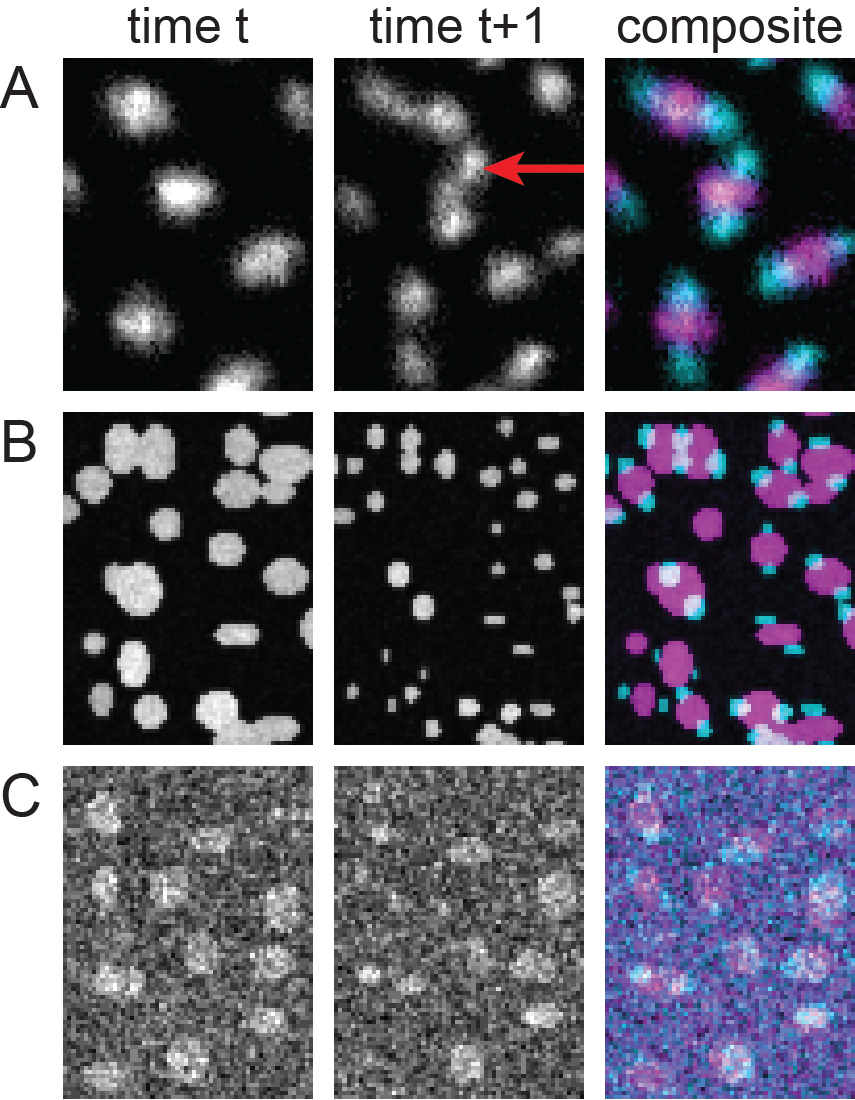

Supplement: Supplementary Data [file supp_bts706_Supplement.zip › Figure_S1.jpg]

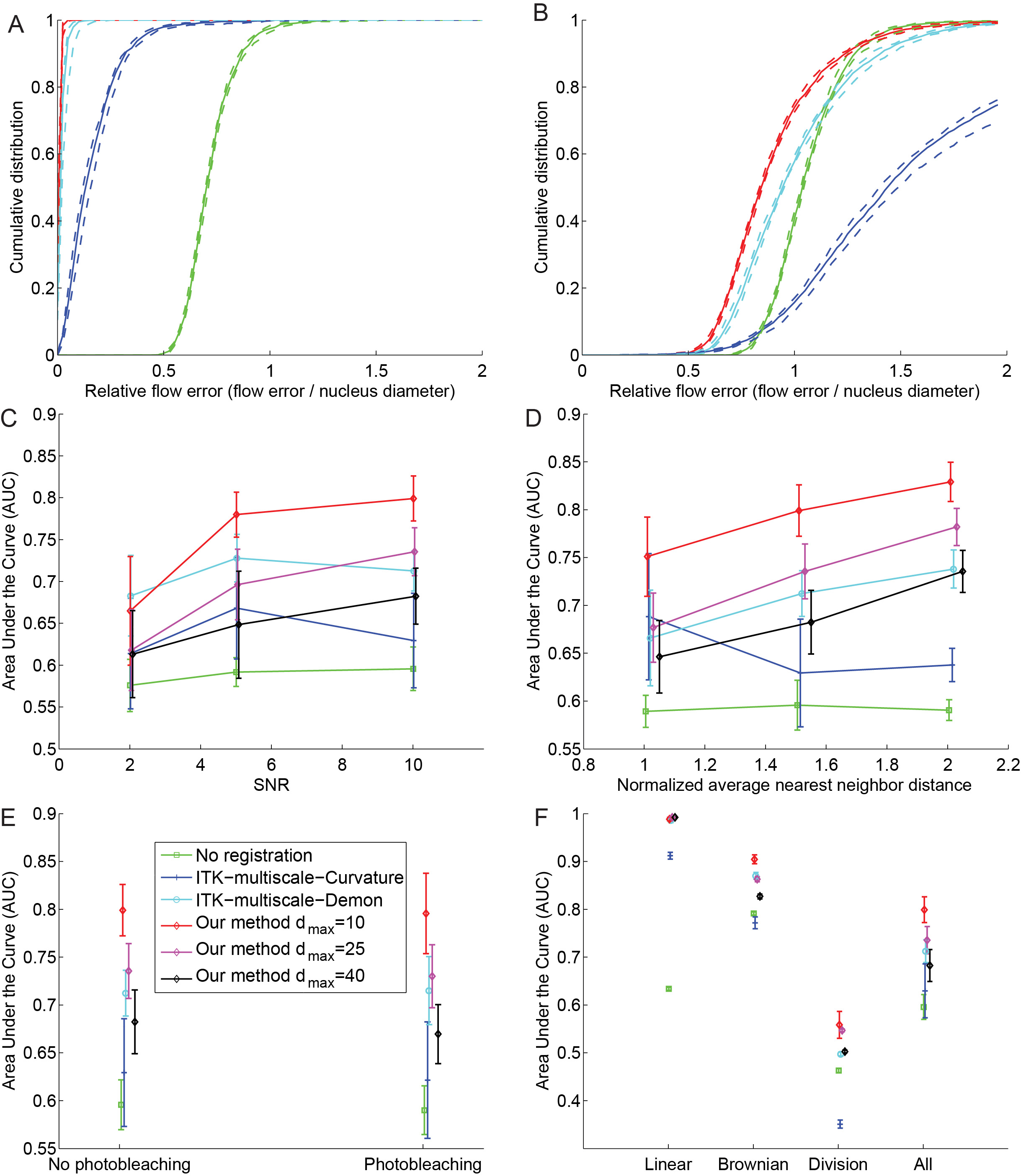

Supplement: Supplementary Data [file supp_bts706_Supplement.zip › Figure_S2.jpg]
